# Increasing the Impact Factor in the Ethical Way

**DOI:** 10.4274/balkanmedj.2017.6.0001

**Published:** 2017-12-01

**Authors:** Cem Uzun

**Affiliations:** 1 Department of Otolaryngology, Trakya University School of Medicine, Edirne, Turkey

The impact factor of a journal is one of the most discussed issues among the new editors of low-tier journals. This is probably because the impact factor is commonly used as one of the best and popular metrics of journal quality (1,2). Thus, an editor who wants to become successful might be evaluated according to her or his journal’s impact factor. However, impact factors are slow to change and, thus, are inappropriate for the evaluation of an editor’s success before the four-year editorial task has ended. Additionally, the impact factor should be used with caution when evaluating an entire journal (3,4).

The impact factor of a journal is calculated by dividing the number of citations received by a journal within a specific year by the citable articles published in that journal in the previous two years (5). The formula for the impact factor is as follows:

Accordingly, editorial improvement (i.e., for the review process) would show up in the next year’s publications. Let’s assume that an editor has been assigned to a present journal in year A. The editor would learn the impact factor of the year (A-1) during their first months of assignment (typically sometime after June when the previous year’s impact factors are published). If the editor makes any changes in the evaluation process, these would affect the publications in the year (A+1) and later. According to the impact factor formula above, editorial improvements would only affect the impact factor of the year (A+3), which is published in the year (A+4) Journal Citation Report.

The primary aim of a good scientific journal should not be to have a high impact factor. Although impact factor is an important journal metric, the main goal of a journal should be to become a high-quality journal. If the scientific quality and the visibility of the articles increase, the impact factor will rise accordingly.

There are four main approaches to increase the quality of a small non-profit scientific journal:

How to increase the quality of a journal?

• Attract quality manuscripts

• Do good editorial work

• Increase visibility of the journal

• Trustful cooperation with owner & publisher

According to my editorial experience at the Balkan Medical Journal as an associate editor (2004-2008) and editor-in-chief (2012-2016); asking authors no charge for submission or publication fee, open access to current issues and archive, having a user-friendly online submission system, fast and appropriate review process and publication, and reaching a large international audience/readers via popular indexes helped the journal to attract many manuscripts. The number of annual submissions to the Balkan Medical Journal has increased more than twenty-fold in the last 10 years and got over 2000. Accordingly, the number of quality manuscripts increased as well, especially those being indexed in popular indexes such as Science Citation Index Expanded (SCIE), PubMed Central (PMC), and Medline.

Good editorial work mainly depends on a compatible, responsible, and motivated editorial board. The important international editor and scientific publication organizations, such as European Association of Science Editors, Council of Science Editors, World Association of Medical Editors, International Committee of Medical Journal Editors, and Committee on Publication Ethics, have defined the rules for a good editorial work. I strongly suggest that new editors read these organizations’ documents; follow their rules; attend their meetings, courses, and workshops; become a member of these organizations; and follow their discussion groups. Additionally, the editor courses given by Pippa Smart and Ana Marusic were very useful. They are not only good teachers but also good people (Figure 1). As a summary, good editorial work includes providing a trustful publication platform; providing an objective review process; having a low acceptance rate; working promptly; providing good professional English editing; providing a statistical review; continuing the education of editors, authors, and reviewers; and following major international editor associations. In addition, short titles with results and a well-structured abstract are important when editing a paper (6,7).

In addition to the major scientific literature search engines (e.g., PubMed and Web of Science) Google is one of the most popular search engines used by academics. A journal’s visibility can be further increased by providing open access to all articles in HTML, PDF and XML format published at the journal’s website; indexing in PMC, and other popular indexes; informative e-mails sent to readers after the publication of new issues; news about the important articles; and publishing good quality review articles.

The improvements made in the editorial process of the Balkan Medical Journal in recent years have increased the journal’s visibility and quality. The principal improvements include a strict review process; standardized statistical and ethical evaluation of all manuscripts before acceptance; professional English editing freely provided to all accepted papers; user friendly online submission system; publication of the journal’s archive at its web page; its open access nature; the educational activities of the journal; and indexing in PMC, resulting in easy and free access to full texts of published articles. However, the success of the journal could not be possible without the efforts of the volunteering professors that make up the editorial board and also the continuing economic support from the Trakya University.

Consequently, the impact factor of the journal has increased more than ten-fold and is currently greater than 1.000 (Figure 2). Since August 2016, I have been serving as an Editor-at-Large at the Balkan Medical Journal, which is now the best general medical journal in Turkey (based on impact factor).

One of my latest evaluations in 2016 revealed that the most citable articles in the Balkan Medical Journal were invited review articles and original articles followed by editorials. Case reports and other articles were cited less. Also, 81% of submissions were from Turkey, with the remaining 19% from 43 different countries. Thus, the international submissions rate should be increased. However, most of the citations to the journal’s articles were made by international authors.

There are also unethical ways to increase the impact factor of a journal (8,9,10), and editors must be aware of and avoid these unacceptable behaviours. Editors must not ask or force authors to cite their journals’ articles. Also, editors must not unnecessarily ask authors to resubmit their valuable original study as a letter, which is not a citable item. Any citations to letters are added to the journal’s total citations, but the denominator does not increase. An editor can suggest a study to be submitted as a letter when there is a scientific reason for that (e.g., small sample size). However, this should not be aimed at increasing the impact factor of a journal. Another unethical way to increase the impact factor is journals’ cooperation (citation stacking), in which journals cite each other, thereby increasing the number of citations (11,12). Journals must also avoid unnecessary self-citations (13,14).

In conclusion, impact factors can be manipulated and have several limitations. The primary aim of a scientific journal should not be having a high impact factor but to become a high-quality journal through appropriate editorial processes and publishing high-quality, ethical articles. If the scientific quality of the articles published in a journal, as well as the visibility of the journal, are increased, the impact factor will rise.

## Figures and Tables

**Figure 1:**
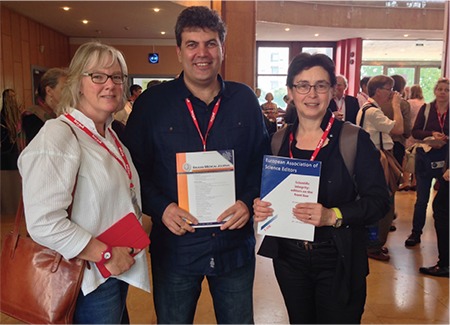
(left to right) Pippa Smart (Editor-in-Chief, Learned Publishing), Cem Uzun (Editor, Balkan Medical Journal), and Ana Marusic (President of EASE) at the EASE 2016 Strasbourg Conference

**Figure 2:**
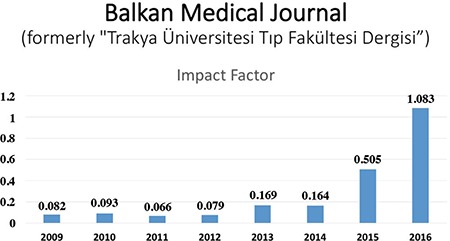
Impact Factor of Balkan Medical Journal since its indexing in Science Citation Index Expanded
